# Crystal structure of a low molecular weight activator Blm-pep with yeast 20S proteasome – insights into the enzyme activation mechanism

**DOI:** 10.1038/s41598-017-05997-4

**Published:** 2017-07-21

**Authors:** Julia Witkowska, Małgorzata Giżyńska, Przemysław Grudnik, Przemysław Golik, Przemysław Karpowicz, Artur Giełdoń, Grzegorz Dubin, Elżbieta Jankowska

**Affiliations:** 1 Faculty of Chemistry, University of Gdańsk, Wita Stwosza 63, 80-308 Gdańsk, Poland; 2 Faculty of Biochemistry, Biophysics and Biotechnology, Jagiellonian University, Gronostajowa 7, 30-387 Krakow, Poland; 3 Malopolska Centre of Biotechnology, Jagiellonian University, Gronostajowa 7, 30-387 Krakow, Poland

## Abstract

Proteasomes are responsible for protein turnover in eukaryotic cells, degrading short-lived species but also removing improperly folded or oxidatively damaged ones. Dysfunction of a proteasome results in gradual accumulation of misfolded/damaged proteins, leading to their aggregation. It has been postulated that proteasome activators may facilitate removal of such aggregation-prone proteins and thus prevent development of neurodegenerative disorders. However, the discovery of pharmacologically relevant compounds is hindered by insufficient structural understanding of the activation process. In this study we provide a model peptidic activator of human proteasome and analyze the structure-activity relationship within this novel scaffold. The binding mode of the activator at the relevant pocket within the proteasome has been determined by X-ray crystallography. This crystal structure provides an important basis for rational design of pharmacological compounds. Moreover, by providing a novel insight into the proteasome gating mechanism, our results allow the commonly accepted model of proteasome regulation to be revisited.

## Introduction

The ubiquitin-proteasome system (UPS) is responsible for degradation of the majority of cytosolic proteins. The central element of this system, 26S proteasome, is composed of a multicatalytic 20S core particle (CP) and a 19S regulator. The role of the latter is to remove degradation marks (deubiquitination) and to unfold the proteins designated for proteolysis within the CP. The eukaryotic CP is a barrel-shaped structure composed of four stacked heptameric rings, each consisting of seven different subunits of either α- or β-type, arranged in the αββα fashion^1, 2^. The luminal chamber formed by these rings accommodates six active sites of three different proteolytic activities: peptidylglutamyl peptide hydrolyzing (PGPH) (β1/β1′ subunits), trypsin-like (T-L; β2/β2′ subunits), and chymotrypsin-like (ChT-L; β5/β5′ subunits) peptidases.

In the latent 20S proteasome, access of polypeptides to the catalytic chamber is restricted by the N-termini of the α subunits, which form a kind of a gate^3^. Apart from ATP-dependent 19S regulators (PAN in *Archaea*), two additional classes of activating proteins may facilitate passage of substrates to the proteasome interior: 11S (also known as PA28 in higher eukaryotes and PA26 in *Trypanosoma brucei*) and Blm10/PA200 (*Saccharomyces cerevisiae*/human). It is postulated that all activating particles display a common mechanism of action which involves anchoring at the proteasome surface and repositioning of the Pro17 reverse turn. This activator binding-induced shift of the Pro17 position results in reorganization of the N-termini of the α subunits, enabling substrate access into the catalytic chamber^4^.

There are only three proteinaceous ligands whose crystal structures in the complex with proteasome are known so far: – Blm10, Pba1-Pba2, and PA26. The first two proteins anchor to proteasome using their C-terminal HbYX motif (Hb – hydrophobic residue, Y – tyrosine, X – any residue), whose binding is believed to enable repositioning of the Pro17 reverse turn and cause the gate opening. Activation by PA26 requires involvement of two regions: the C-terminal one, deficient of the HbYX motif, is needed for binding to proteasome, while the so-called activation loop is responsible for the Pro17 shift and opening the entrance to the catalytic chamber^5^. PA26 is a heptameric protein and as such may create contacts with multiple intersubunit pockets: the electron density of the C-termini of PA26 has been detected in α2/α3, α3/α4, α4/α5, and α5/α6 pockets in complexes with yeast and archeal proteasomes^6^. Monomeric Blm10 interacts exclusively with only one, the α5/α6 pocket^4^. Its C-terminal carboxylate forms a salt bridge with the conserved α6Lys62 (equivalent to Lys66 in archeal proteasome) whereas a side chain of the penultimate tyrosine interacts with α5Gly19, prompting translocation of the α5Pro17 reverse turn. Such an interaction significantly affects only a single *α* subunit and thus the entrance pore in Blm10-20S complex is only partially open^4, 7^. In multimeric activators, like PA26, binding affects multiple *α* subunits resulting in wider opening of the entrance gate^8^.

Quite recent cryo-EM structures of human and yeast 26S proteasomes revealed some details about the interactions of the catalytic 20S core and 19S regulator^9–12^. Three subunits of 19S, namely Rpt2, Rpt3 and Rpt5, possess the HbYX motif. In human 26S two of them – Rpt3 and Rpt5 – were found to bind in the proteasome intersubunit pockets, Rpt3 in α1/α2 and Rpt5 in α5/α6^9, 11^. Nevertheless, the gate leading to the catalytic chamber remained closed in this structure. A similar situation was observed in the case of y26S, where binding of three subunits with the HbYX moiety did not trigger the gate opening^10, 12^. The open conformation was observed, however, in one conformational state of y26S, in which the fourth subunit – Rpt6 – was bound within the proteasome α ring^12^. The other known examples of the proteasome with the gate open are crystal structures of complexes of archeabacterial 20S with mutated PA26. In these constructs either (i) seven or eight residues of the C-terminal sequence of PA26 were substituted with the sequence of PAN’s C-terminus, holding the conserved HbYX motif (PDB: 3JTL/3IPM, respectively)^8, 13^; or (ii) only the penultimate Val residue of PA26 was exchanged into Phe (PDB: 3JSE) or Tyr (PDB: 3JRM)^8^. In all these structures multiple contacts of the binding motifs with the proteasome intersubunit pockets were observed, resulting in the displacement of all seven Pro17 clusters and the gate opening.

The proteasome plays an important role in numerous homeostatic and regulatory processes, including cell cycle progression, growth and atrophy of tissues, and oncogenesis^14–16^. By degrading mutated, misfolded or oxidatively damaged proteins the proteasome is involved also in cellular quality control^17^. Defects in proteasome function play an important role in the pathophysiology of a number of disorders, including inflammation, autoimmune and neurodegenerative diseases, and various cancers. Its broad implication in pathological processes makes the proteasome a suitable target for pharmacological intervention. Many competitive small-molecule inhibitors of the proteasome have been developed, and three have been approved by the FDA for use in blood cancer treatment: bortezomib, carfilzomib and ixazomib^18, 19^. In contrast, development of proteasome activators has been almost neglected, mainly because of a poor understanding of the underlying mechanisms, including a lack of structural explanation and definition of binding sites. It was only recently that stimulation of the proteasome was proposed in management of Parkinson’s and Huntington’s diseases^20, 21^ and oxidative stress consequences^22–24^. To facilitate progress in this field, in this study we have focused on characterization of a new proteasome activator: Blm-pep. It is a 14-residue peptide, which can be a starting point for bioisosteric replacements providing more stable and efficient activators. We designed this compound based on mechanistic features of the Blm10 protein. The significance of particular segments of Blm-pep in its ability to activate human ChT-L, T-L and PGPH peptidases has been evaluated using a number of analogs. A crystal structure of Blm-pep in a complex with yeast proteasome has been obtained and revealed a closed conformation of the enzyme entrance pore, which is consistent with the lack of activity of Blm-pep against y20S. A molecular dynamics simulation was undertaken to explain the observed different capacity of Blm-pep to activate orthologous human and yeast proteasomes. The obtained results allow us to propose corrections to the currently established model of the proteasome gating mechanism.

## Methods

### Synthesis of Blm peptides

The peptides were synthesized on either TentaGel R PHB or Wang resin (loading capacity of 0.2 or 0.35 mmol/g, respectively). Syntheses were performed using a Microwave Liberty Blue synthesizer (CEM) or Millipore model 9050 Plus peptide synthesizer. The first amino acid was attached to the resin using a symmetrical anhydride or 1-(2-mesitylenesulfonyl)-3-nitro-1H-1,2,4-triazole/1-methylimidazole method, and the coupling efficiency was determined by measurement of absorbance of the resulting fulvene-piperidine adduct, at λ = 301 nm. Subsequent residues were added using standard Fmoc/tBu chemistry. Crude peptides were purified to at least 98% purity by reversed-phase high-performance liquid chromatography (RP-HPLC) using a Jupiter Proteo column (21.2 × 250 mm, 4 μm, 90 Å; Phenomenex). A linear gradient of acetonitrile in 0.1% aqueous trifluoroacetic acid (TFA) or acetonitrile in 0.1 M triethylamine phosphate buffer, pH 3.0, was used as a mobile phase. The purity of the synthesized compounds was evaluated by analytical RP-HPLC using a XB-C18 Aeris Peptide column (4.6 × 150 mm, 3.6 μm, 100 Å, Phenomenex) and a 30 min linear gradient of 5–80% acetonitrile in 0.1% aqueous TFA. UV absorption was monitored at λ = 223 nm. The molecular weight of the peptides was confirmed by matrix-assisted laser desorption/ionization time-of-flight (MALDI TOF) mass spectrometry or electrospray ionization ion trap time-of-flight liquid chromatography mass spectrometry (ESI IT TOF LCMS) with a C12 Jupiter Proteo column (150 × 2 mm, 4μm, 90 Å; Phenomenex). MS data are listed in Supplementary Table 1S.

### Proteasome activity assays

The influence of the peptides on the catalytic activities of the 20S proteasome was tested using latent housekeeping CP isolated from either human erythrocytes or yeast cells (Enzo Life Sciences). The CP was utilized at a final concentration of 2 nM. The following fluorogenic peptide substrates were employed to determine the activity of proteasomal peptidases: succinyl-Leu-Leu-Val-Tyr-4-methylcoumarin-7-amide (Suc-LLVY-MCA, for determination of ChT-L activity), *tert*-butyloxycarbonyl-Leu-Arg-Arg-4-methylcoumarin-7-amide (Boc-LRR-MCA, for T-L activity), and carbobenzoxy-Leu-Leu-Glu-4-methylcoumarin-7-amide (Z-LLE-MCA, for PGPH activity). Stock solutions of the substrates and the tested peptides were prepared in dimethyl sulfoxide (DMSO). To minimize any unspecific effect of DMSO its concentration was kept below 3% of the final reaction volume. All assays were performed at 37 °C in the 96-well plate format using a reaction volume of 100 μl. Tests were carried out in 50 mM Tris-HCl (pH 8.0, measured at 20 °C). The peptides were tested in the concentration range of 0.05–10 μM. Substrates were added at 100 μM final concentration. The release of aminomethylcoumarin (AMC) was monitored continuously for 60 min by measuring fluorescence at 460 nm (Infinite 200 PRO, TECAN) at 2-min intervals. All activity assays were performed at least in triplicate, each as an independent measurement, utilizing separate aliquots of the modulator, substrate and enzyme stock solutions. The peptidolytic activity was calculated as nanomoles of the released AMC product per milligram of CP per second. In the case of the control this value was regarded as 100% activity of the latent proteasome. Values for the tested compounds were determined in an identical way and calculated as a percentage of the control.

### Proteolytic stability assay

Blm-pep was incubated either with human or yeast 20S proteasome. Both assays were conducted in 25 mM Tris-HCl buffer (pH 8.0, measured at 20 °C), which for y20S contained 0.25 mM EDTA as an additive. The peptide and proteasome concentrations in 100 μl final solution were 10 µM and 0.025 mg/ml, respectively. Incubation was carried out for 3 hours at 37 °C. The reaction was stopped by addition of 5 μl of 10% TFA. Results were analyzed using RP-HPLC and MALDI TOF. Prior to MS analysis the samples were desalted using C18 spin columns (Pierce).

### Isolation and purification of the yeast 20S proteasome

For crystallization experiments, 20S proteasome was isolated from *Saccharomyces cerevisiae* (strain MHY501) according to previously published protocols^25–27^. In brief, *S. cerevisiae* were cultured in YPD (1% yeast extract, 2% peptone, 2% glucose) to OD_600nm_ > 1. Cells were collected by centrifugation and lysed in a continuous cell disruption system in lysis buffer (50 mM Tris-HCl pH 7.5, 250 mM sucrose, 1 mM DTT). The lysate was clarified by centrifugation and the proteasome-containing fraction was recovered by centrifugation at 120 000 x g. The pellet was resuspended in 50 mM Tris-HCl pH 7.5 containing 20% of glycerol and applied to a Pierce Strong Anion Exchange Spin Column (29.7 × 122 mm, Thermo Scientific). The column was washed with a step gradient of NaCl (up to 2 M), and the proteasome eluted at 400 mM NaCl. The protein content in the collected fractions was verified based on activity tests against the proteasome ChT-L substrate. Active fractions were pooled and further purified using a hydroxyapatite type II column (CHT type II, Bio Rad) with increasing phosphate gradient (Na_2_HPO_4_/NaH_2_PO_4_ pH 7.5, 20% glycerol). The proteasome eluted at approximately 220 mM phosphate buffer. The final polishing step consisted of gel filtration on Superose 6 in 50 mM Tris/HCl pH 7.5.

### Crystallization

Just prior to crystallization, 20S proteasome was concentrated to 2.5–5 mg/ml using Amicon Ultra 100 K centrifugal concentrators (Merck Millipore). Crystals were grown by the hanging drop vapor diffusion method at 20 °C, as previously described^28^. Drops were prepared by adding 0.5 µl of protein stock, 0.4 µl of reservoir solution (35 mM magnesium acetate, 13% MPD, 0.1 M MES pH 6.5) and 0.1 µl of 4% DMSO as an additive. Crystals appeared within 7 days and continued to grow for about two weeks. The preformed crystals were soaked for 24 hours with Blm-pep (0.5 µM) and flash frozen after cryoprotection in 25% ethylene glycol in the mother liquor.

### Structure determination and refinement

Diffraction data were collected at 100 K at the 14.1 beamline at the Helmholtz Zentrum in Berlin, Germany. The data was indexed and integrated using XDS^29, 30^ and scaled using the SCALA program contained in the CCP4 package^31^. The crystal structure was determined by molecular replacement using MOLREP^32^ and coordinates of the apo 20S yeast proteasome (PDB ID: 1RYP) as a search model. Initial refinement was performed using Refmac 5.024^33^ while Phenix^34^ was used in the later stages. Torsion-angle noncrystallographic symmetry (NCS) averaging was applied. Five percent of reflections were used for the cross-validation analysis and the progress of refinement was monitored using the R_free_ parameter^35^. The model was constructed in Coot^36^. Blm-pep ligand was introduced at the final stages of refinement into the clearly defined electron density. The quality of the final structure was assessed using a MolProbity server^37^. Data collection and refinement statistics are summarized in Table 2S. The structure was deposited at the Protein Data Bank (PDB ID: 5NIF). The final models were analysed and figures were prepared using Coot and PyMol (Delano Scientific, USA).

### Molecular modeling

Structures of human constitutive 20S proteasome (PDB ID: 4R3O) and yeast 20S proteasome in complex with Blm-pep determined in this study (PDB ID: 5NIF) were used in modeling. The C-terminal HbYX motif anchors Blm-pep between the α5 and α6 subunits of yeast proteasome, and it was modeled in the same place for the human counterpart. To obtain the lowest energy structure the model was minimized (500 cycles) and optimized by short, low temperature (2 ps, 50 K) molecular dynamics in repetitive cycles using the AMBER v.12 package^38^. This procedure allowed the constructed model to correspond to the experimental data to the maximum possible extent. The model of Blm-pep binding to human proteasome was analyzed using the RasMol AB program^39^.

## Results

### Design of Blm-pep activator

Blm-pep (*K* **YF**TGS*K* LWRSYYA) was designed based on the Blm10 activator of 20S proteasome and other prior data. To provide an anchor at the α subunit surface of the proteasome we used a sequence derived from the C-terminus of Blm10 (LWRSYYA), which provides such interactions. Our earlier studies^40, 41^ revealed the importance of basic amino acids for proteasome allosteric modulation and therefore we incorporated two lysine residues into the design of Blm-pep. Tyrosine and phenylalanine residues were further incorporated into Blm-pep with the intention of providing aromatic contacts with the N-termini of the α subunits, which we believed are important in the mechanism of proteasome gate opening (for a more detailed explanation of the basis of Blm-pep design, see Supplementary information). A polar linker (TGS) was additionally added to improve solubility and elongate the peptide to allow concomitant anchoring at the intersubunit pocket and interactions with the residues surrounding the entrance to the catalytic channel.

### Proteolytic stability of Blm-pep

A proteolytic stability assay revealed that Blm-pep is digested by both human and yeast 20S proteasome (Supplementary Information, Fig. 1S). To determine cleavage sites, digestion reactions were analyzed by mass spectrometry. Some differences between the degradation patterns of yeast and human proteasomes were spotted, although they were not significant.

### Activity of Blm-pep and delimitation of the active fragment

**Figure 1:**
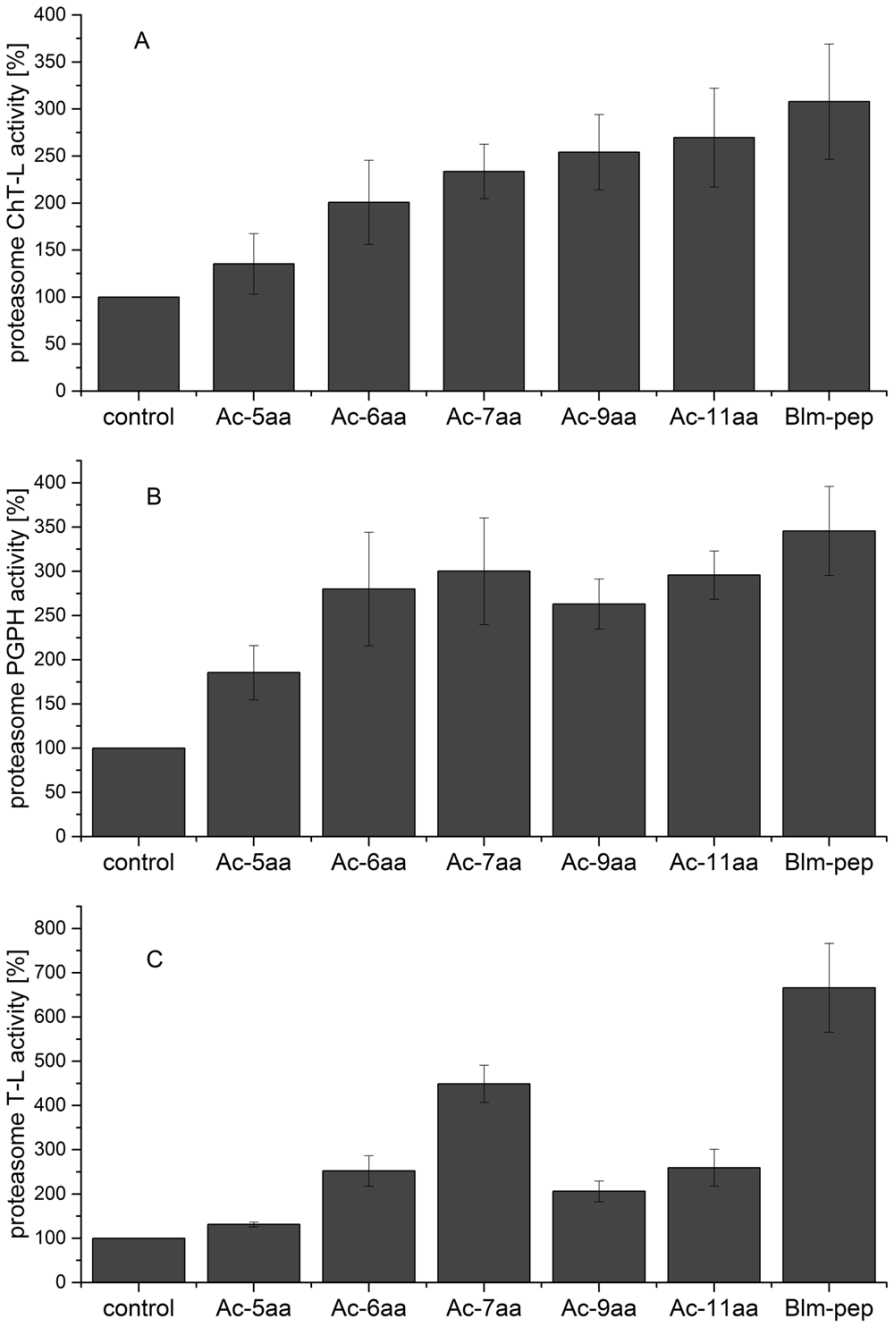
Activation of different proteolytic activities of the latent human 20S proteasome by Blm-pep and its acetylated analogs: (**A**) ChT-L, (**B**). PGPH, (**C**). T-L peptidase activity. The peptides concentration was 10 μM. Results are expressed as a percentage of activity of the latent human 20S proteasome. The variability of the data is presented as standard deviation error bars.

Activity assays demonstrated that Blm-pep efficiently stimulated the activity of human 20S proteasome in a dose-dependent manner (Supplementary Information, Fig. 2S). Interestingly, three distinct activities of this multienzyme were differently stimulated. At 10 μM concentration of Blm-pep, ChT-L and PGPH activities increased around three times whereas T-L activity increased around seven-fold (Fig. 1). Surprisingly, Blm-pep did not influence any of the activities of yeast 20S proteasome at the concentrations sufficient to activate h20S.

To test the role of particular fragments of Blm-pep in human proteasome activation, we synthesized shorter analogs, consisting of 5, 6, 7, 9 and 11 amino acid residues (Table 1).

**Table 1:** Sequences of Blm-pep and its shorter analogs. MS data are included in Supplementary Table 1S.

Peptide	Sequence
Blm-pep	KYFTGSKLWRSYYA
Ac-5aa	Ac-RSYYA
Ac-6aa	Ac-WRSYYA
Ac-7aa	Ac-LWRSYYA
7aa	LWRSYYA
Ac-9aa	Ac-SKLWRSYYA
9aa	SKLWRSYYA
Ac-11aa	Ac-TGSKLWRSYYA
11aa	TGSKLWRSYYA

The ability of the tested analogs to stimulate ChT-L peptidase activity increased linearly with the length of the analog (Fig. 1A). Whereas Ac-5aa only slightly affected the activity of ChT-L (a 1.3-fold increase), the activity of Ac-11aa was only slightly lower than that observed for Blm-pep. Such a correlation between peptide length and capability to stimulate h20S was not observed for PGPH peptidase: all tested Blm-pep analogs, except for the shortest one (Ac-5aa), increased the activity of PGPH by around 2.5–3-fold, which is comparable to Blm-pep (Fig. 1B). The activation of T-L peptidase by the tested peptides was less consistent. Ac-6aa and Ac-7aa enhanced the activity 2.5- and 4.5-fold, respectively, compared to almost 7-fold activation obtained with Blm-pep (Fig. 1C). Concomitantly, the longer fragments, Ac-9aa and Ac-11aa, both stimulated T-L activity only less than 3-fold.

**Figure 2:**
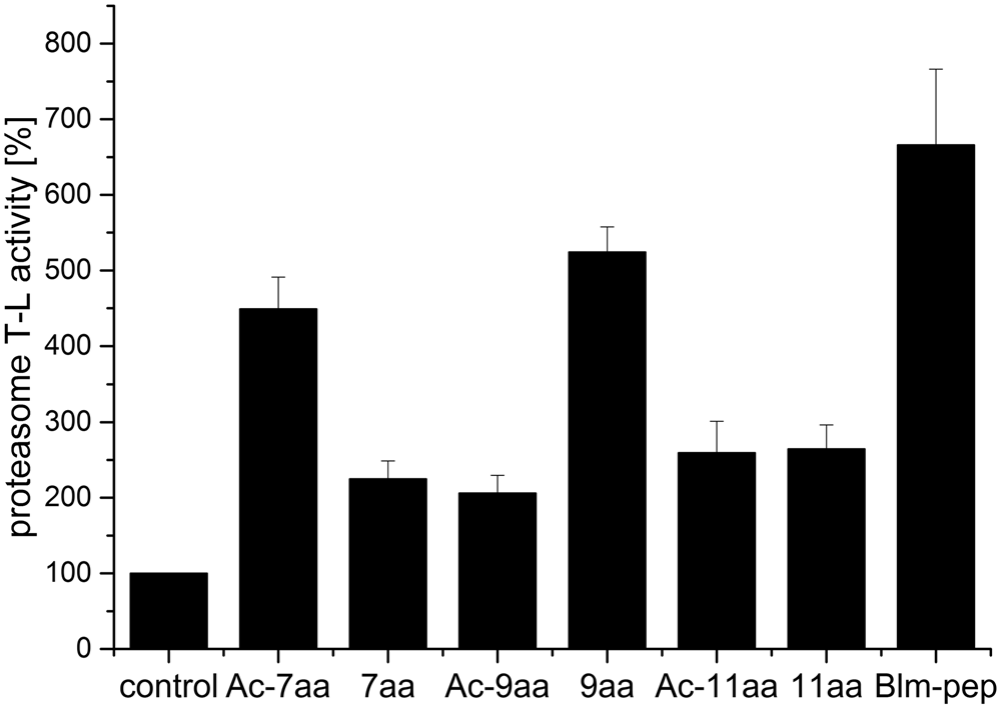
Stimulating potential of acetylated and non-acetylated Blm-pep analogs towards T-L peptidase of the latent human 20S proteasome. The peptides concentration was 10 μM. Results are expressed as a percentage of activity of the latent human 20S proteasome. The variability of the data is presented as standard deviation error bars.

All the above discussed Blm-pep fragments were acetylated to preserve peptide bond-like structure at their *N*-termini. To evaluate the influence of a free *N*-terminal amine group, present in the parent Blm-pep activator, we additionally tested three non-acetylated analogs. No influence of *N*-terminal acetylation was observed in the case of ChT-L activation (Supplementary Information, Fig. 3S). In the case of PGPH peptidase certain small differences between the activity of acetylated and nonacetylated peptides were noted, although they were not significant (Supplementary Information, Fig. 2S). In contrast to the above, acetylation significantly influenced the activation capability of certain peptides towards T-L peptidase (Fig. 2). The 9aa fragment was twice as potent as Ac-9aa in stimulating T-L activity. The opposite effect was observed for the 7aa peptide, whose activity was significantly lower compared to Ac-7aa. In the case of the eleven-residue fragment no influence of acetylation was observed on its capability to stimulate T-L peptidase.

Neither of the acetylated or nonacetylated analogs influenced the activity of yeast 20S proteasome, consistent with the lack of activity of Blm-pep towards this protease.

### Structural basis of the Blm-pep-proteasome interaction

To elucidate the structural basis of the activating effect of Blm-pep we ventured to crystallize the peptide in a complex with both human 20S proteasome and yeast proteasome. Crystals were obtained for both proteins, however they diffracted sufficiently only in the second instance, despite significant optimization effort.

The crystal structure of the yeast proteasome:Blm-pep complex was determined at 3 Å resolution. The crystals belonged to the P21 space group and contained a single proteasome molecule in the asymmetric unit. The structure was solved by molecular replacement using a model of yeast constitutive proteasome as a probe (PDB ID: 1RYP). The structure was refined with R_work_ and R_free_ values of 0.17 and 0.23, respectively, and was characterized by decent geometry. The crystallographic data collection and refinement statistics are summarized in Supplementary Information Table 2S.

**Figure 3:**
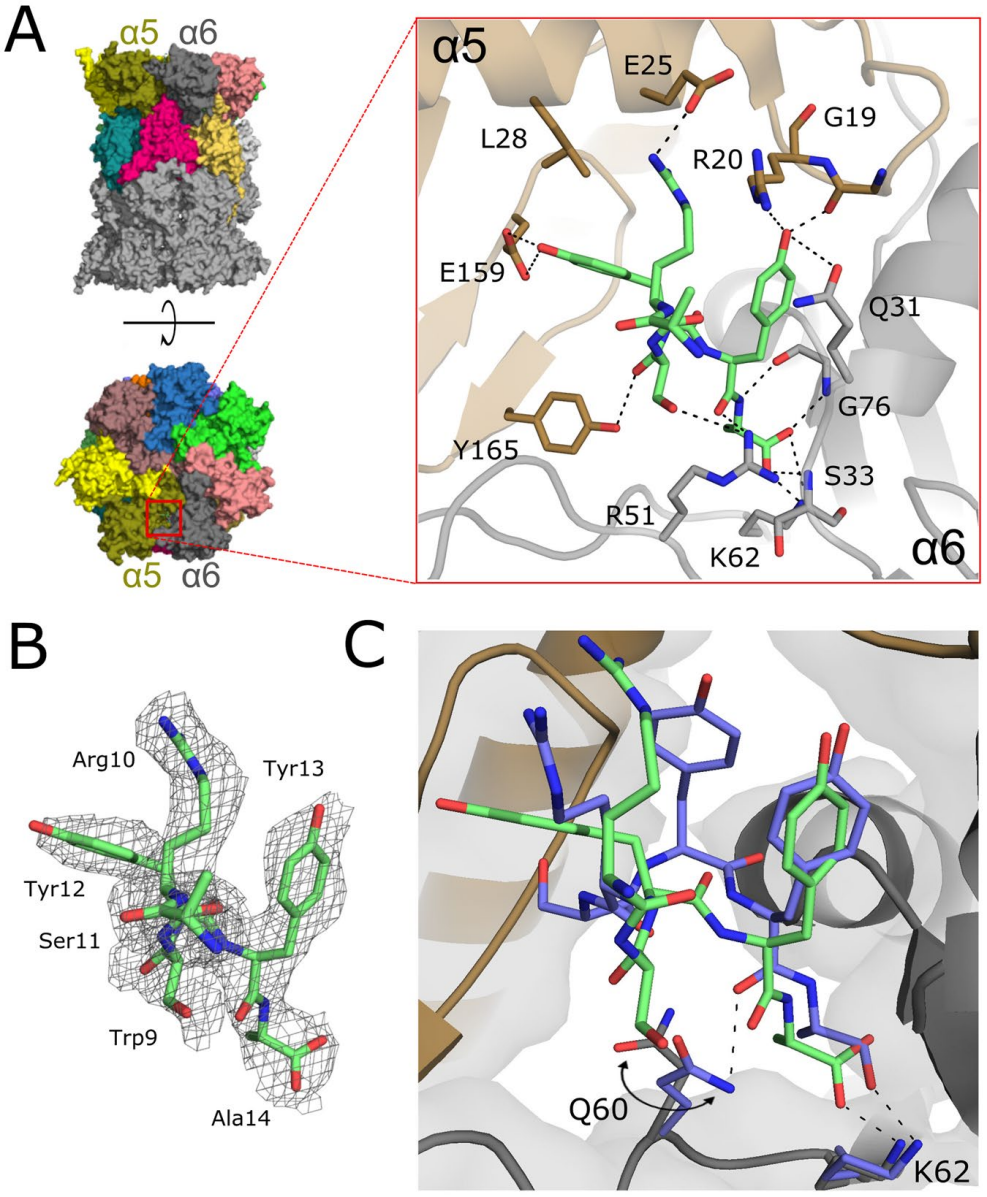
Interaction of Blm-pep with yeast 20S proteasome. (**A**) General localization of Blm-pep binding site between subunits α5 and α6 and detailed interactions guiding Blm-pep binding (blow-up). (**B**) Electron density defining Blm-pep fragment included in the model (2Fo-Fc omit map contoured at 1σ level). (**C**) Comparison of the binding modes of Blm-pep (green) and Blm10 (blue) at the surface of yeast 20S proteasome (the difference in interaction of both activators with Q60 is highlighted).

The electron density accounting for a part of the activator was clearly defined in two pockets, between α5 and α6 and, symmetrically, the α5′ and α6′ subunits, even prior to the introduction of the molecule into refinement (Fig. 3B). Despite relatively low resolution, this density unambiguously defines the five C-terminal amino acids (10–14) of Blm-pep (Fig. 3A). The rest of the peptide is not defined by electron density, accounting for the flexibility of this part and lack of stable interactions with the proteasome.

Blm-pep anchors deep into a pocket between the α5 and α6 proteasome subunits at the face of the channel gate (Fig. 3A). The C-terminus of Blm-pep is buried at the very bottom of the pocket and is stabilized by hydrogen bonds with the side chain amine of α6Lys62 and the main chain amides of α6Ser33 and α6Gly76. Ala14 is further stabilized by a hydrogen bond between its main chain amide and the α6Gly76 carbonyl. The main chain carbonyl of Tyr13 participates in one hydrogen bond - with α6Arg51, whereas its side chain provides multiple contacts, mainly through its hydroxyl group, which forms hydrogen bonds with the main chain nitrogen of α5Arg20, the main chain carbonyl of α5Gly19 and the side chain amide of α6Gln31. Both Tyr13 and Tyr12 also participate in hydrophobic interactions: Tyr13 with the side chain of α6Gln31, and Tyr12 with α5Leu28. Apart from this, the side chain hydroxyl of Tyr12 participates in a hydrogen bond with the α5Glu159 carboxylate. The main chain carbonyl of Ser11 is involved in a hydrogen bond with the hydroxyl group of α5Tyr165. The side chain of Arg10 forms a salt bridge with α5Glu25. The main chain of Trp9 points out of the binding pocket into the solvent region, not contributing any interactions with either α5 or α6 subunits, and the side chain of this residue is already poorly defined by electron density and was not included in the final model. Such a binding mode is very closely followed in the two symmetrical pockets (the full list of the contacts at the both proteasome α faces is provided in Table 3S included in Supplementary Information). Further residues are not defined by electron density, suggesting transient or no interaction with y20S.

The molecular details of Blm10 interaction with yeast proteasome have been elucidated previously^4^. Since Blm-pep was partially designed based on Blm10, it was interesting to compare the interactions of those two molecules with yeast proteasome. Blm-pep binds at the same α5/α6 intersubunit pocket as the C-terminal part of Blm10, however the interactions guiding the affinity of the two molecules remain considerably different (Fig. 3C). The ultimate alanine residue is oriented almost identically in both structures and its affinity is guided by the same primary canonical interaction of the terminal carboxyl group and the side chain amine of α6Lys62 and backbone amides of α6Ser33 and α6Gly76, further stabilized by the interaction of the backbone amide and the carbonyl oxygen of α6Gly76. The overall disposition of the penultimate tyrosine residue and the interactions provided by its side chain are comparable in both structures, but the binding of the backbone is already guided by different interactions. In Blm10 the backbone carbonyl participates in a hydrogen bond with the side chain of α6Gln60, whereas such an interaction is not observed in the structure containing Blm-pep, due to the different disposition of the side chain of α6Gln60. Beyond the penultimate tyrosine, the disposition and, consequently, the interactions involving Blm10 and Blm-pep are completely different in the two structures (Fig. 3C). In particular, Blm-pep does not follow the β-sheet-like hydrogen bond pattern observed in Blm10. Overall, despite the fact that the C-terminal part of Blm-pep was designed based on Blm10, the binding modes of the two activators differ quite considerably.

**Figure 4:**
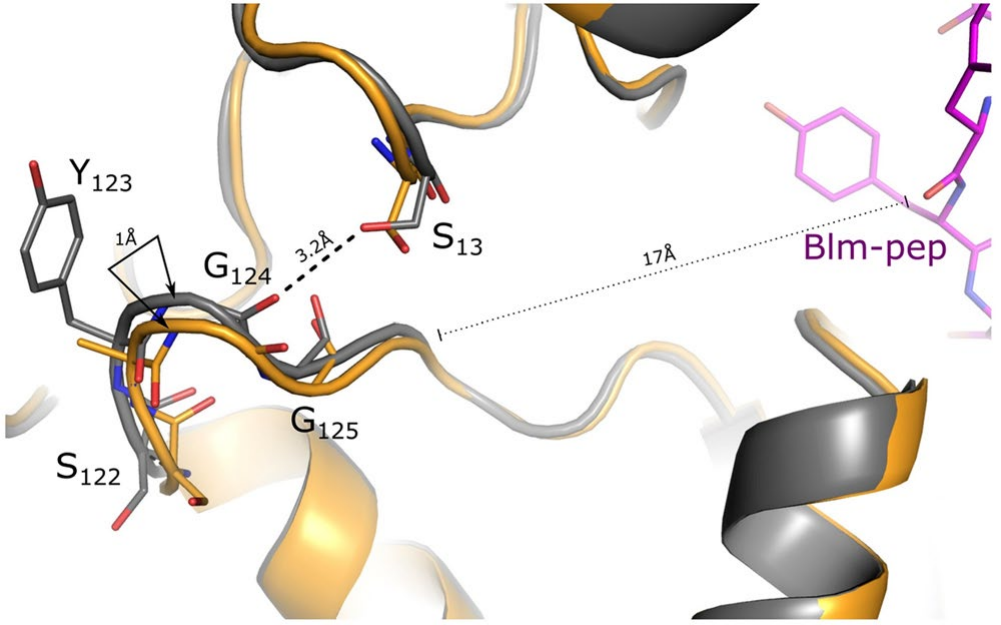
Distal conformational change of the 20S induced by binding of Blm-pep. The change involves the highly conserved loop, which is a part of the α-annulus regulating the substrate access to the catalytic center. Color coding is as follows: grey - latent proteasome (1RYP); beige – the proteasome bound to Blm-pep.

Upon binding of Blm10 the proteasome β-subunits do not move substantially (RMSD 0.4 Å), but the α-subunits move somewhat to form a partially open entrance pore^4^. In the structure of the y20S:Blm-pep complex the enzyme is found in its closed conformation, which supports the fact that Blm-pep failed to activate y20S. Nevertheless, local alignment of the Cα atoms of the β-rings of our structure with that of the modulator-free y20S results in RMSD values of 0.6 Å and 0.9 Å for the β- and α-rings, respectively. The progressive increase in RMSD from the core region to the distal ends of the proteasome indicates that although Blm-pep binding does not induce gate opening it causes small changes in the architecture of the α-ring, presumably resembling the initial stages of gate opening. Such a small change can be detected in the position of the highly conserved Tyr123-Gly124-Gly125 loop, which is a key element of the aperture (α-annulus) restricting the access of substrates to the catalytic center in the latent proteasome^42^. Upon Blm-pep binding this loop shifts more than 1 Å, which results in breaking of a hydrogen bond between the Gly124 carbonyl and the hydroxyl group of Ser13 (Fig. 4). Since Blm-pep binds to 20S in two copies only, this shift is limited to the α6/6′ subunits and does not cause a rotation propagating around the whole α-ring, as it was observed in archeal 20S interacting with PAN peptides^43^. Nevertheless, the Blm-pep:y20S complex is characterized by a conformation which can be called the intermediate between that of the free – closed, and partially opened proteasomes.

### Model of Blm-pep:h20S interaction

Despite great effort, we were unfortunately unable to obtain crystals of sufficient quality to determine the structure of human proteasome with Blm-pep. However, the α5/α6 pockets in yeast and human enzymes show enough similarity to allow creation of a sensible model of Blm-pep:h20S interaction. In fact, the only differences at the binding site include α5Leu28 which is substituted by Ile in h20S, and α6Ala78 which is Thr in h20S. Therefore, we constructed an interaction model by docking Blm-pep, extracted from our experimental structure, to the experimental structure of human constitutive 20S proteasome (PDB ID: 4R3O), and minimizing the interaction energy.

**Figure 5:**
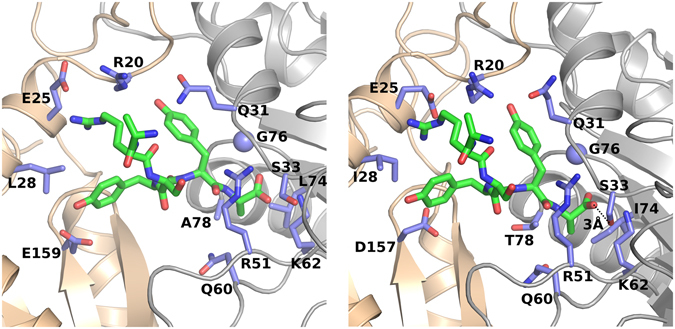
Comparison of Blm-pep interaction with yeast and human proteasome. Left – structure of yeast 20S proteasome (α5 and α6 are colored beige and gray, respectively) in complex with Blm-pep (green), determined in this study (5NIF). Interacting residues within the proteasome are highlighted blue. Right – modeled interaction of Blm-pep with human 20S proteasome (model based on 4R3O; orientation and colour coding same as in the left panel). The hydrogen bond distinguishing the interactions of Blm-pep with yeast and human 20S is shown (see text).

Our model allows us to conclude that interactions of Blm-pep at the α5/α6 pocket of yeast and human proteasome should be largely similar, although some minor differences are also noted (Fig. 5). In both complexes Arg10 forms a salt bridge with α5Glu25. The interactions of Tyr12 with α5Leu28/α5Ile28 and α5Glu159/α5Asp157, respectively, in yeast/human proteasome, are comparable in their nature. In addition, the interactions of Tyr13 with α5Arg20, α6Arg51 and α5Gly19 are conservative in both yeast and human proteasomes. The salt bridge of the C-terminal carboxylate of Blm-pep and α6Lys62 is again conservative, as are two further hydrogen bonds connecting the carboxylate with the backbone of α6Ser33 and α6Gly76. However, the interaction of the C-terminal carboxylate with the side chain of α6Ser33 is possible only in the human proteasome. The latter hydrogen bond constitutes the only apparent difference in the binding modes of Blm-pep in the α5/α6 pocket of human and yeast proteasomes.

## Discussion

Efficient activators of human 20S proteasome might alleviate the deleterious consequences of certain pathogenic states by stimulating degradation of damaged proteins and preventing their aggregation into toxic oligomers. Such a strategy could potentially find utility in treatment of Parkinson’s and Huntington’s diseases^20, 21^ or management of oxidative stress consequences^22–24^. Nevertheless, development of proteasome activators is hindered by insufficient structural understanding of the activation process. To facilitate the design of proteasome modulators we determined the structure of the y20S proteasome in a complex with Blm-pep, a peptide capable of stimulating the three proteolytic activities of human 20S. This crystal structure demonstrates for the first time the mode of binding of a low molecular weight activator at atomic resolution. All previously available information on proteasome activation was either based on proteinaceous activators or did not reach high resolution and indicated only the sites at which an activator had been found, which did not enable detection of its specific interactions with the proteasome.

**Figure 6:**
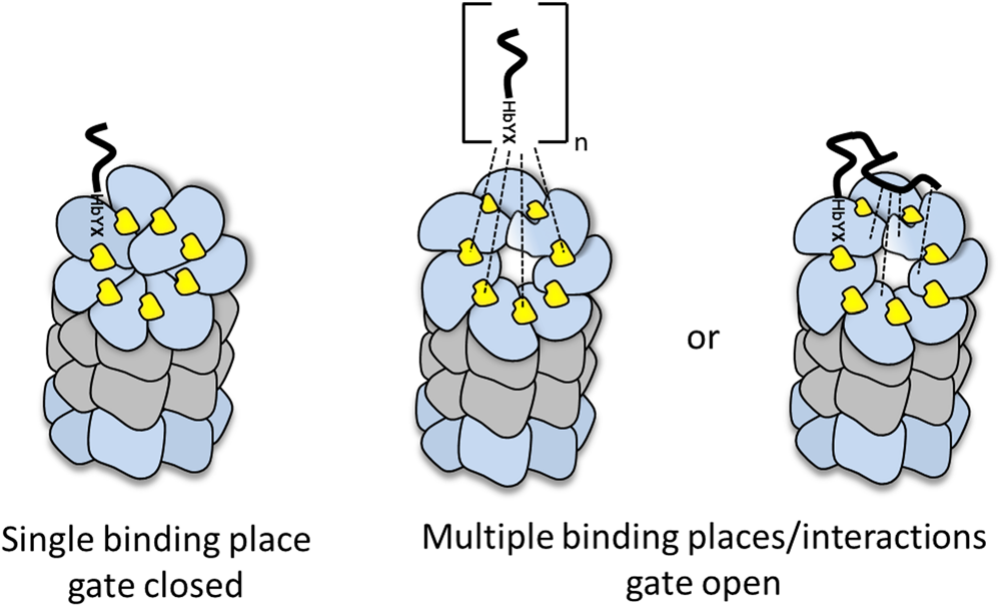
Revisited model of proteasome activation supporting all currently available data. Binding of a sigle HbYX motif is not sufficient to allow substrate access. Only interaction at multiple HbYX binding sites or at a single site together with secondary interactions allow for efficient proteasome activation.

Our structure revealed that Blm-pep occupies a single site at each proteasome α-ring and defined the molecular details of the interaction. The binding pocket is located between the α5/α6 subunits. The same pocket is occupied by the C-terminus of Blm10^4^, a proteinaceous activator on which we based the design of our peptide activator. Interestingly, the overall interactions guiding the affinity of Blm-pep and Blm10 are not identical, despite both molecules sharing the C-terminal sequence responsible for their anchoring to the proteasome α-ring. Moreover, Blm10 induces a partially open conformation of the entrance pore whereas the pore is closed in the structure of the complex of y20S with Blm-pep. Such an observation is corroborated by the fact that Blm-pep was unable to activate the proteolytic activity of yeast proteasome. Nevertheless, the closed conformation of the proteasome in the complex with Blm-pep is not identical to the closed conformation of the unliganded proteasome. In fact, it may be described as an intermediate between the unliganded and Blm10-bound structure. This indicates that although binding of the HbYX motif at the α-ring may be necessary for induction of an open conformation, as demonstrated by others^6, 13, 44^, single HbYX motif binding is not sufficient, and only induces an intermediate, but still closed conformation. It now seems clear that there must be secondary interactions which help Blm10 to partially open the gate. In dissecting the interface between Blm10 and 20S we found another region in the proteasome α-face which is in close contact with the activator. It encompasses the N-termini of the α-subunits, which in the unliganded proteasome pack tightly against each other, stabilizing the closed conformation. In the crystal structure of the proteasome complex with Blm10, only the α5 and α6 N-termini – which create the pocket hosting the binding motif^4, 37^ – are clearly defined in the electron density maps. These N-termini apparently reorient upon ligand binding, pointing upwards into the cavity within the ligand body (Fig. 4S). Conformation of the α5 and α6 subunits is stabilized due to a number of contacts with the ligand residues, other than its C-terminal HbYX (Table 4S). This new finding about the possible importance of secondary interactions requires the classical model of proteasome activation to be revisited. It had been believed thus far that proteasome activation by a protein partner possessing a singular HbYX motif is driven by binding of this motif at the intersubunit pocket, thereby reorienting the Pro17 reverse turn and inducing the open conformation of the gating pore. In the light of the above discussed data it is more probable that binding of a single HbYX motif induces a pre-open state, but is insufficient to result in sustained gate opening. Only secondary interactions outside the α pocket would stabilize a fully open conformation in the case of monomeric activators (Fig. 6, right).

It has been demonstrated that the HbYX motif-holding C-terminal peptide derived from PAN stimulates proteasome activity by inducing an open conformation of the entrance gate of *T. acidophilum* proteasome^43^. This, however, does not contradict our conclusions since the α-ring of archeal proteasome is build of identical subunits and therefore the peptide activator may bind in all seven pockets. In eukaryotic proteasomes α-subunits are not identical and y20S binds Blm-pep at only a single site. We hypothesize that in the case of small activators, such as Blm-pep, in which interactions at secondary sites are unlikely, only a collaborative effect of interactions at multiple sites is capable of sufficiently stabilizing the open conformation (Fig. 6, center). Such a hypothesis is corroborated by the fact that two different C-terminal peptides of the 19S regulator show a cooperative effect: while Rpt5 is able to activate the proteasome, conjoint additon of Rpt2 increases the activity several times^45^. Our hypothesis is further supported by the recently published cryo-EM structure of the 19S:h20S complex, which showed a closed conformation of the entrance pore despite the C-termini of two HbYX-holding subunits of 19S regulator being found deeply immersed in the enzyme’s intersubunit pockets^11^. Moreover, in a similar 3D reconstruction of yeast 26S, binding of three HbYX-possessing subunits of 19S was also insufficient to render the gate permanently opened^12^. Concomitantly, in the cryo-EM structure of *T. acidophilum* proteasome soaked with a 7-residue peptide derived from the C-terminal sequence of PAN, the structural organization of the gating pore was different and the entrance pore notably wider than in the closed form^43^. In this structure, however, the ligand molecules were found in all seven pockets of the α-ring, and also in all seven proteolytic sites located in the β-ring. Although direct demonstration is still necessary, all these results advocate that only concominant action of multiple HbYX motif-containing peptides may result in a fully open conformation and therefore an efficient activator must occupy more then a single intersubunit pocket. Alternatively, the activator must provide secondary interactions with the gating residues. Such a conclusion has important implications for the design of novel activators which must be flexible enough to overcome interpocket variance present in the eukaryotic proteasome.

Although Blm-pep is quite an efficient stimulator of human 20S peptidases it did not display capability to activate the yeast proteasome. A similar lack of activating power has been detected by Dange *et al*. in the case of an 8-residue peptide derived from the C-terminal sequence of Blm10, which did not activate the ChT-L peptidase of y20S^40^. It was also reported that this peptide stimulated T-L and PGPH activities, but this was not the case for Blm-pep, which did not show any activating potency toward the yeast enzyme peptidases. We do not know, however, what concentration of a modulator was used in the studies of Dange *et al*. since this information was not disclosed in their paper.

Our hypothesis on the collaborative action of small molecule activators might explain why Blm-pep activates only human 20S proteasome, but not its yeast ortholog. Comparison of the intersubunit pockets of both enzymes demonstrates that some pockets of the yeast proteasome are less spacious than their human counterparts and may be more selective and exclude some ligands. Thus, it is possible that a lack of opportunity for collaborative action of activator molecules is responsible for the fact that Blm-pep does not activate yeast 20S proteasome, although it activates its closely related human ortholog. It is also of note that the assymetric α-ring of eukaryotic proteasomes renders the α2, α3, α4 and to a lesser extent α5 subunits special positions in closing the entrance to the catalytic chamber^46^. Binding of activator molecules in the pockets created by these subunits may be an indispensable element of the proteasome activation mechanism. The pockets located between these subunits are empty in our closed-gate structure. In the structures of 19S regulator with human or yeast 20S, in which the proteasome gate is also closed, only the α1/α2 and α5/α6 pockets, or the α1/α2, α3/α4 and α5/α6 pockets, are occupied, respectively^11, 12^. In contrast, in the PA26:y20S complex, in which the electron density of PA26 C-termini were detected in α2/α3, α3/α4, α4/α5 and α5/α6 pockets, the proteasome gating pore is fully open^37^. In addition, in the s4 proteolytic state of y26S, described by Wehmer *et al*., the gate was found open when the fourth – α2/α3 – intersubunit pocket became involved in interactions with Rpt subunits of the 19S regulator, joining the α1/α2, α3/α4 and α5/α6 pockets, which are occupied in the closed and pre-open conformations of the s1-s3 states^12^.

Only 5 residues of Blm-pep are defined by electron density in our structure, and they are located at the α-pocket of the proteasome. At the same time, longer variants of Blm-pep demonstrated higher activity compared to shorter ones. This suggests that the extended N-terminal part of Blm-pep provides additional transient interactions at the gating channel. Moreover, the fact that the influence of N-terminal acetylation on the compound activity depends on the length of the activator further advocates the presence of transient interactions within the N-terminal part. Interactions of the basic N-terminal amine group may help in initiation of gate opening, on condition, however, that it reaches a suitable partner. If not, it may even obstruct positive conformational changes, disabling activation of the proteasome, which we observed in the case of the 7aa analog as compared to Ac7aa (Fig. 2). Such transient contacts, even if present, are not amenable to crystallographic analysis and are highly speculative at this point.

In conclusion, we have characterized the structural basis of the interaction of a low molecular weight proteasome activator with the target protease, facilitating rational design of more drug-like activators. Our hypothesis on the collaborative function of intersubunit pockets in proteasome activation may have important implications for the design of pharmacologically relevant molecules, though this still requires better experimental validation.

## Electronic supplementary material

Supplementary Information
