# Supplementary material for: Crystal structure of a low molecular weight activator Blm-pep with yeast 20S proteasome – insights into the enzyme activation mechanism
Source: Sci Rep. 2017 Jul 21;7:6177. doi: 10.1038/s41598-017-05997-4 (PMC5522460; doi:10.1038/s41598-017-05997-4)
Supplement: Supplementary file 1 — Supplementary Information [file 41598_2017_5997_MOESM1_ESM.pdf]

Crystal structure of a low molecular weight activator Blm-pep with yeast 20S proteasome – insights into the enzyme activation mechanism

Julia Witkowska[1], Małgorzata Giżyńska[1], Przemysław Grudnik[2,3], Przemysław Golik[2,3], Przemysław Karpowicz[1], Artur Giełdoń[1], Grzegorz Dubin[2,3*], Elżbieta Jankowska[1*]

[1] Faculty of Chemistry, University of Gdańsk, Wita Stwosza 63, 80-308 Gdańsk, Poland
[2] Faculty of Biochemistry, Biophyiscs and Biotechnology, Jagiellonian University, Gronostajowa 7, 30-387, Krakow, Poland
[3] Malopolska Centre of Biotechnology, Jagiellonian University, Gronostajowa 7, 30-387, Krakow, Poland

[*] corresponding authors:
elzbieta.jankowska@ug.edu.pl
grzegorz.dubin@uj.edu.pl

Table 1S. Mass spectrometry characterization of Blm-pep and its analogs.

peptide	sequence	m/z	
		observed	calculated
Blm-pep	KYFTGSKLWRSYYA	1771.7	1770.0
Ac-5aa	Ac-RSYYA	701.3	700.7
Ac-6aa	Ac-WRSYYA	887.1	887.0
Ac-7aa	Ac-LWRSYYA	1000.4	1000.1
7aa	LWRSYYA	957.9	958.0
Ac-9aa	Ac-SKLWRSYYA	1215.4	1215.4
9-aa	SKLWRSYYA	1173.9	1173.3
Ac-11aa	Ac-TGSKLWRSYYA	1372.6	1373.5
11-aa	TGSKLWRSYYA	1332.3	1331.6

Rationale for Blm-pep design – incorporation of basic and aromatic residues

Analysis of electrostatic potential of the α surface in 20S proteasome shows that its central part is rather acidic and may prefer basic residues in the interacting ligands.

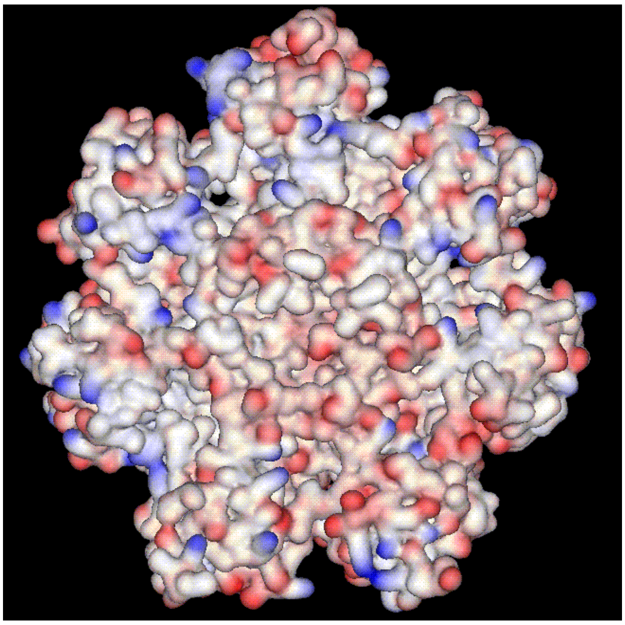

Electrostatic potential of proteasome α surface (yeast proteasome 20S, PDB code: 1RYP)

In most structures of 20S proteasome complexed with its proteinaceous activators the N-termini of α subunits are not visible so it is difficult to detect changes in their conformation upon the activator binding. In the structure of Blm10:y20S complex the α5 and α6 subunits, which create the binding place for the ligand C-terminus, are visible starting from the first residue. It can be seen in this structure that the N-terminus of α6 subunit points upwards upon Blm10 binding and forms an aromatic interactions with the ligand residue.

A. B. C.

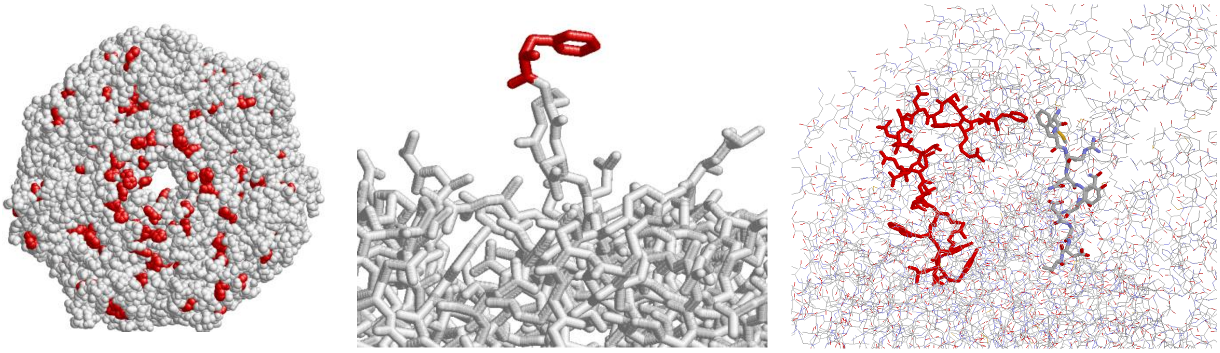

A. Aromatic residues (red) surround the entrance to the proteasome catalytic channel, B. Pointing upwards the N-terminus of proteasome α6 subunit with Phe2 residue marked in red. The proteasome structure presented in panels A and B is extracted from its complex with PA26 (PDB code: 1FNT), C. Complex of proteasome with Blm10 (PDB code: 3L5Q). The same α6Phe2 residue points upwards upon binding to Blm10, and makes an aromatic contact with Phe2120 of the activator. The C-terminal residues of the activator sequence and the N-terminus of α6 subunit are presented as sticks, the activator residues are marked in red.

Table 2S. Crystallographic data statistics for the yeast 20S proteasome:Blm-pep activator complex.

**Crystallographic data**	
wavelength (Å)	0.918
resolution range (Å)	50.01 - 3.0 (3.107 - 3.0)*
space group	P 1 21 1
unit cell<br>dimensions (Å)<br>angles (°)	 a=134.044, b=302.01, c=143.84<br>90 112.55 90
unique reflections	210039
completeness (%)	99.93 (99.90)*
I/σ(I)	5.25 (1.69)*
Wilson B-factor	43.69
R-merge (%)	11.17 (45.05)*
**Refinement statistics**	
Reflections used in refinement	209976 (20925)*
Reflections used for R_{free}	10362 (1032)*
R_{work}	0.1743 (0.2722)*
R_{free}	0.2270 (0.3389)*
No. of non-hydrogen atoms	49080
protein	48888
ligand	126
water	66
average B-factor (Å^2)	39.47
rmsd bond lengths (Å)	0.009
rmsd bond angles (°)	1.07
Ramachandran favored (%)	95.02
Ramachandran allowed (%)	4.68
Ramachandran outliers (%)	0.30

* - data in parentheses are for the outermost / highest resolution shell

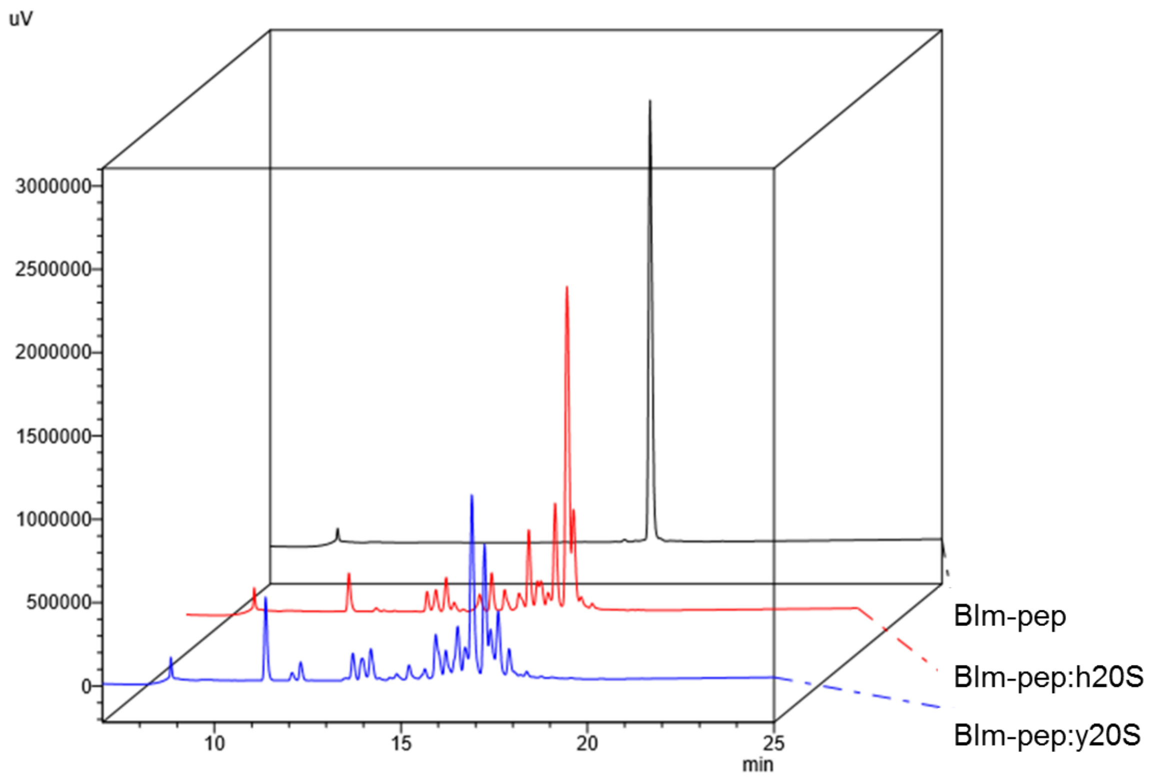

Figure 1S. HPLC chromatograms showing proteolytic degradation of Blm-pep by yeast (blue) and human (red) 20S proteasomes. Chromatogram of Blm-pep is shown as a black line.

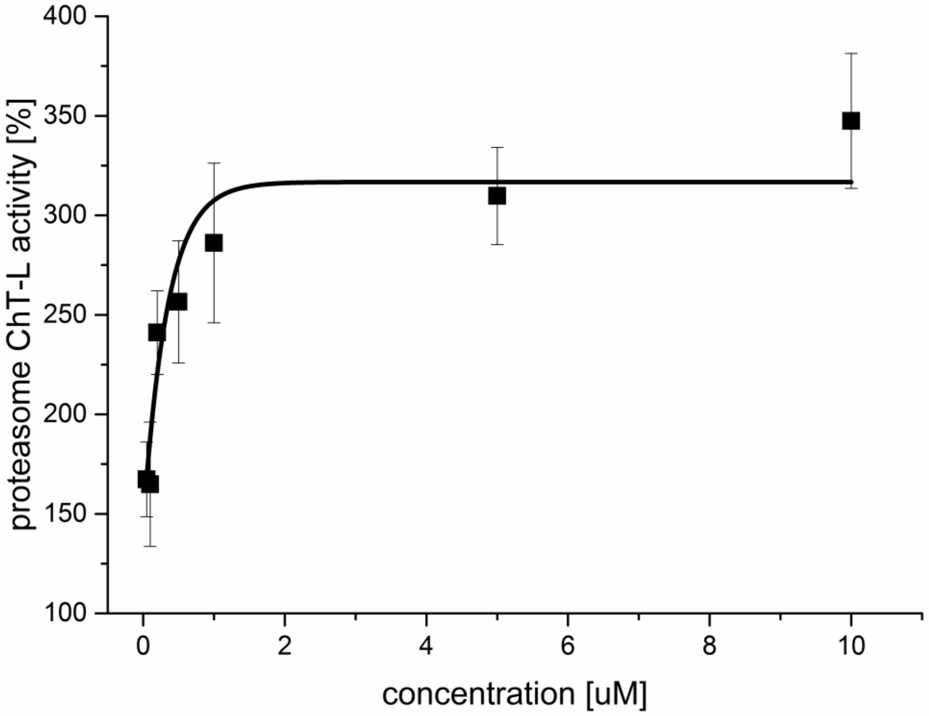

Figure 2S. Titration of the latent human 20S proteasome by Blm-pep. The presented results are mean values of at least three measurements. The variability of the data is presented as standard deviation error bars. Curve fitting was performed using nonlinear (asymptotic) regression (R-square = 0.923).

We have also tested higher concentrations of Blm-pep, 100 and 200 μM. At these concentrations, however, Blm-pep inhibited h20S. Such an effect is not surprising since allosteric activators can inhibit their target enzymes/receptors at higher doses, probably because they start to compete with substrates.

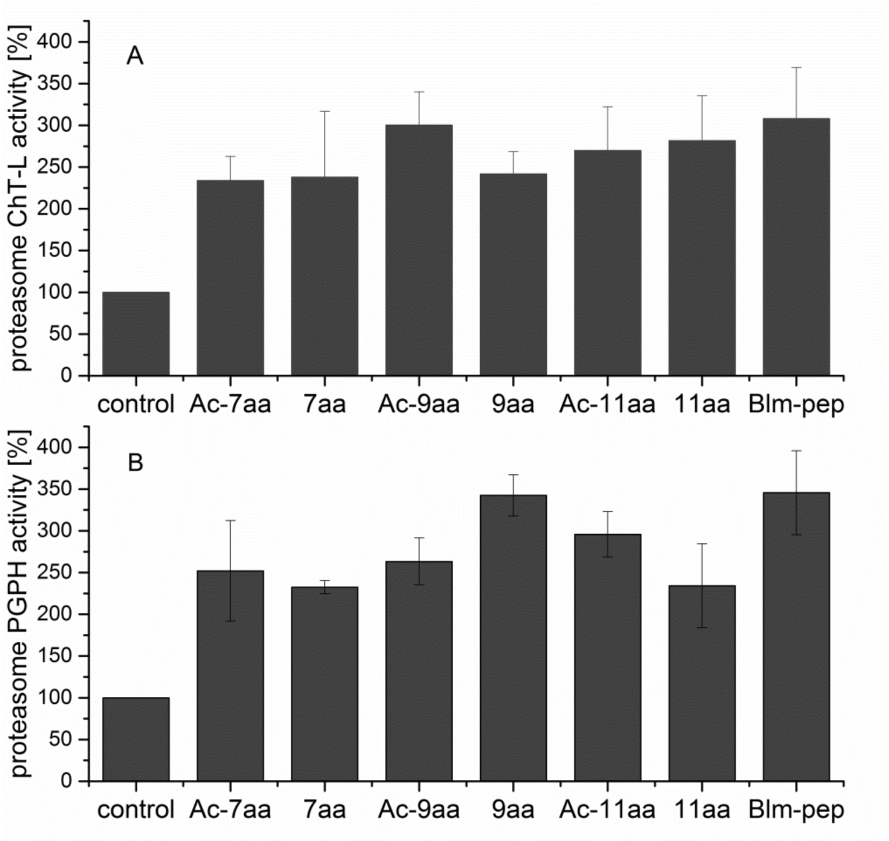

Figure 3S. Comparison of stimulating capability of acetylated and non-acetylated Blm-pep analogs towards: A. ChT-L, and B. PGPH peptidases of the latent human 20S proteasome. The peptides concentration was 10 μM. Results are expressed as a percentage of activity of the latent human 20S proteasome. The variability of the data is presented as standard deviation error bars.

Table 3S. The list of hydrogen bonds created by Blm-pep residues with the proteasome α (subunits E and F) and α’ (subunits S and T) faces. These contacts were generated by CCP4 program.

Proteasome residues	Blm-pep residues	Hydrogen bonds [Å]	
		subunits	
		EF	ST
α5 20(ARG) / NE [N]:	10(ARG) / NE [N]	3,07	3,71
α5 20(ARG) / NE [N]:	10(ARG) / NH2 [N]		3,2
α5 20(ARG) / NE [N]:	10(ARG) / NH1 [N]		3,21
α5 20(ARG) / NH1[N]:	10(ARG) / NH2 [N]		3,3
α5 20(ARG) / NH2[N]:	10(ARG) / NE [N]		3,9
α5 20(ARG) / NH2[N]:	10(ARG) / NH2 [N]		3,9
α5 25(GLU) / OE1[O]:	10(ARG) / NH1 [N]	3,44	3,26
α5 25(GLU) / OE1[O]:	10(ARG) / NH2 [N]	2,74	2,63
α5 25(GLU) / OE1[O]:	10(ARG) / NE [N]	3,98	
α5 25(GLU) / OE2[O]:	10(ARG) / NH1 [N]		2,79
α5 25(GLU) / OE2[O]:	10(ARG) / NH2 [N]		3,55
α6 31(GLN) / NE2[N]:	10(ARG) / O [O]	3,97	
α6 51(ARG) / NH1[N]:	10(ARG) / NE [N]		3,98
α5 165(TYR) / OH [O]:	11(SER) / O [O]	3,67	2,96
α5 159(GLU) / OE1[O]:	12(TYR) / OH [O]		3,21
α5 159(GLU) / OE2[O]:	12(TYR) / OH [O]	3,55	3,11
α5 19(GLY) / O [O]:	13(TYR) / OH [O]	2,69	2,85
α5 20(ARG) / N [N]:	13(TYR) / OH [O]	3,73	
α6 31(GLN) / OE1[O]:	13(TYR) / OH [O]	3,85	3,76
α6 31(GLN) / NE2[N]:	13(TYR) / OH [O]		3,66
α6 51(ARG) / NE [N]:	13(TYR) / OH [O]	3,98	
α6 51(ARG) / NH1[N]:	13(TYR) / O [O]	3,13	3,58
α6 33(SER) / N [N]:	14(ALA) / O [O]	3,47	3,3
α6 33(SER) / N [N]:	14(ALA) / OXT [O]	3,13	3,31
α6 62(LYS) / NZ [N]:	14(ALA) / OXT [O]	3,41	3,62
α6 62(LYS) / NZ [N]:	14(ALA) / O [O]		2,57
α6 76(GLY) / N [N]:	14(ALA) / OXT [O]	2,97	2,96
α6 76(GLY) / O [O]:	14(ALA) / N [N]	3,01	3,11

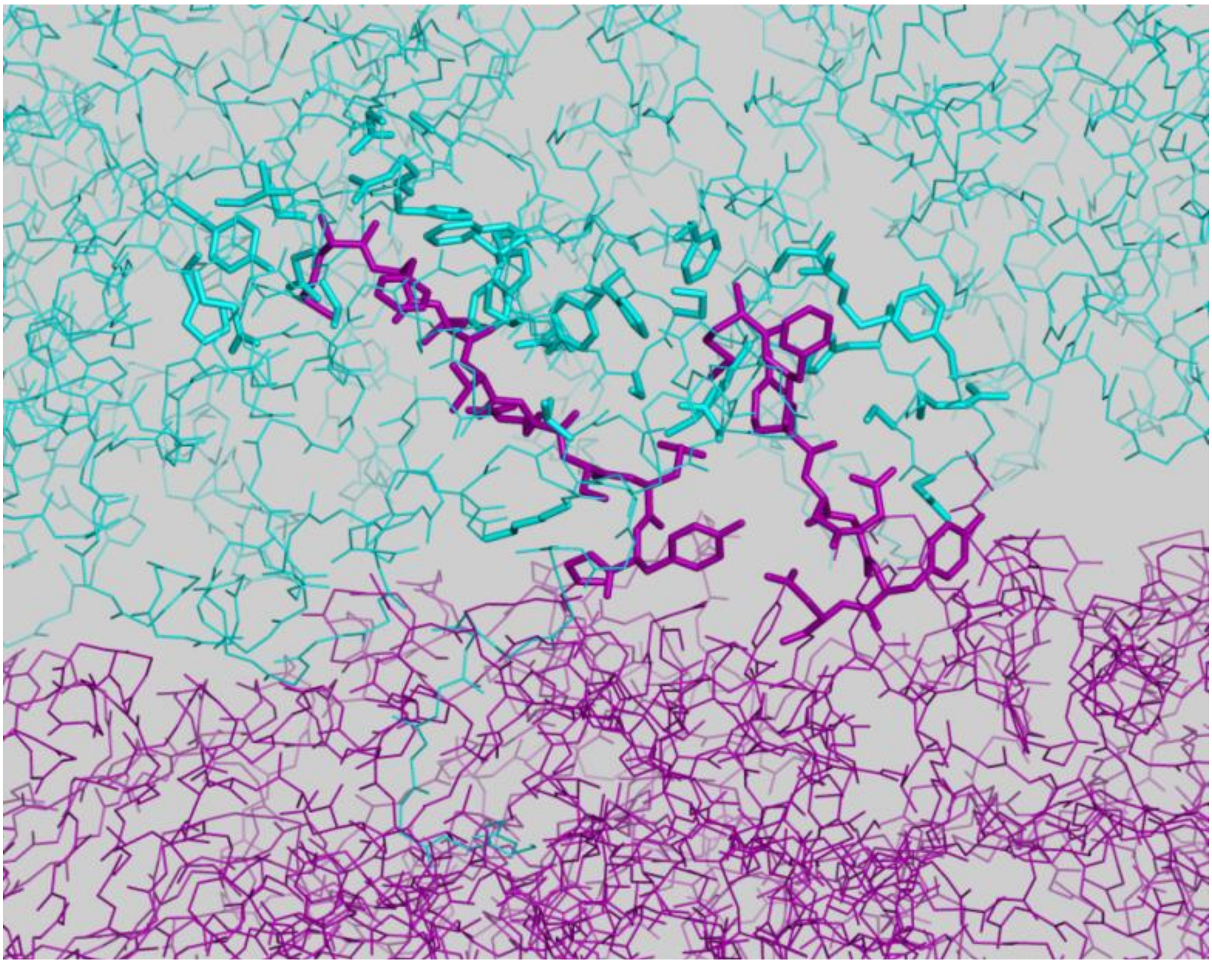

Figure 4S. Interactions of Blm10 (cyan) with α subunits of yeast 20S proteasome (purple) in the neighborhood of the enzyme's entrance pore (PDB: 3L5Q). The N-terminal regions of α5 and α6 subunits and interacting with them residues of Blm10 are displayed as sticks. The C-terminus of Blm10, immersing deeply into the pocket between the α5 and α6 subunits, is also visible.

Table 4S. The list of hydrogen bonds created by Blm10 with the most N-terminal residues of proteasome α subunits, found by CCP4 program based on the crystal structure of Blm10:y20S complex (PDB code: 3L5Q). Numbering of proteasome subunits is as follows: J - α5, K - α6, L - α7.

**Proteasome residues**			**Blm10 residues**			**Distance [Å]**
/1/J/5001(MET)	O	[O]:	480(ARG)	NE	[N]:	3.95
/1/J/5003(LEU)	N	[N]:	520(THR)	OG1	[O]:	4.00
/1/J/5005(ARG)	O	[O]:	525(SER)	OG	[O]:	3.72
/1/J/5007(GLU)	OE2	[O]:	525(SER)	OG	[O]:	3.90
/1/J/5003(LEU)	O	[O]:	526(ASN)	ND2	[N]:	3.88
/1/J/5005(ARG)	NH1	[N]:	527(ASN)	O	[O]:	3.46

/1/K/6003(ARG)	NH2	[N]:	587(GLN)	O	[O]:	2.58
/1/K/6003(ARG)	NH2	[N]:	588(ASN)	O	[O]:	3.95
/1/J/5007(GLU)	OE1	[O]:	589(LYS)	NZ	[N]:	3.74
/1/J/5007(GLU)	OE2	[O]:	589(LYS)	NZ	[N]:	3.11
/1/K/6003(ARG)	NH2	[N]:	589(LYS)	N	[N]:	3.77
/1/K/6004(ASN)	OD1	[O]:	633(ASN)	O	[O]:	3.57
/1/K/6005(ASN)	N	[N]:	633(ASN)	O	[O]:	3.42
/1/K/6005(ASN)	ND2	[N]:	633(ASN)	O	[O]:	3.81
/1/K/6014(SER)	OG	[O]:	633(ASN)	OD1	[O]:	3.35
/1/K/6016(THR)	OG1	[O]:	633(ASN)	OD1	[O]:	3.69
/1/K/6018(ARG)	NH1	[N]:	633(ASN)	ND2	[N]:	3.95
/1/K/6003(ARG)	N	[N]:	634(SER)	OG	[O]:	3.99
/1/K/6003(ARG)	O	[O]:	634(SER)	OG	[O]:	2.47
/1/K/6005(ASN)	OD1	[O]:	635(ARG)	N	[N]:	3.07
/1/L/7005(THR)	OG1	[O]:	635(ARG)	NH1	[N]:	3.83
/1/K/6001(MET)	O	[O]:	636(HIS)	NE2	[N]:	2.97
/1/K/6003(ARG)	NE	[N]:	636(HIS)	ND1	[N]:	3.24
/1/K/6003(ARG)	NH1	[N]:	636(HIS)	ND1	[N]:	3.13
/1/K/6003(ARG)	NH2	[N]:	636(HIS)	ND1	[N]:	2.89
/1/K/6003(ARG)	N	[N]:	636(HIS)	NE2	[N]:	3.59
/1/K/6001(MET)	O	[O]:	637(ARG)	NE	[N]:	3.96
/1/K/6001(MET)	N	[N]:	637(ARG)	NH1	[N]:	3.49
/1/K/6001(MET)	O	[O]:	637(ARG)	NH1	[N]:	3.25
/1/L/7015(SER)	OG	[O]:	676(ASN)	OD1	[O]:	2.81
/1/L/7016(PRO)	N	[N]:	676(ASN)	OD1	[O]:	3.74
/1/L/7017(ASP)	OD1	[O]:	676(ASN)	ND2	[N]:	2.89
/1/L/7017(ASP)	N	[N]:	676(ASN)	OD1	[O]:	3.22
/1/L/7017(ASP)	OD1	[O]:	676(ASN)	OD1	[O]:	2.59
/1/L/7019(ARG)	NH1	[N]:	676(ASN)	ND2	[N]:	3.32
/1/L/7007(TYR)	OH	[O]:	677(ASP)	N	[N]:	3.90
/1/J/5001(MET)	N	[N]:	2016(ASP)	O	[O]:	3.66
/1/J/5003(LEU)	O	[O]:	2074(TYR)	OH	[O]:	3.35
/1/J/5005(ARG)	N	[N]:	2074(TYR)	OH	[O]:	3.02
/1/J/5005(ARG)	O	[O]:	2074(TYR)	OH	[O]:	3.45
/1/J/5020(ARG)	NH1	[N]:	2108(SER)	O	[O]:	3.59
/1/J/5020(ARG)	NH1	[N]:	2108(SER)	OG	[O]:	3.24
/1/J/5020(ARG)	NH2	[N]:	2108(SER)	OG	[O]:	3.01
/1/J/5018(GLU)	OE2	[O]:	2111(LYS)	NZ	[N]:	3.74
/1/J/5006(SER)	OG	[O]:	2112(LYS)	O	[O]:	3.43
/1/J/5009(ASP)	OD2	[O]:	2112(LYS)	NZ	[N]:	3.97
/1/K/6001(MET)	SD	[S]:	2116(ASP)	OD2	[O]:	3.62
/1/K/6002(PHE)	N	[N]:	2116(ASP)	OD2	[O]:	3.20
/1/J/5019(GLY)	O	[O]:	2142(TYR)	OH	[O]:	2.71
